# Perceived Impact of Epilepsy and Its Treatment on Pediatric Patients and Their Families

**DOI:** 10.3390/children12020228

**Published:** 2025-02-13

**Authors:** Redab Al-Ghawanmeh, Ala’a F. Al-Shaikh, Salma Burayzat, Ruba M. Jaber, Mohammad Al-Tamimi, Osama Zidan, Samah Aburahmeh

**Affiliations:** 1 Department of Pediatrics, Faculty of Medicine, The Hashemite University, Zarqa 13133, Jordan; 2 Ministry of Health, Amman 11118, Jordan; 3 Department of Family and Community Medicine, School of Medicine, University of Jordan, Amman 11942, Jordan; 4 Department of Microbiology, Pathology and Forensic Medicine, Faculty of Medicine, The Hashemite University, Zarqa 13133, Jordan; 5 Faculty of Medicine, The Hashemite University, Zarqa 13133, Jordan; 6 Department of Neuroscience, Faculty of Medicine, Jordan University of Science and Technology, Irbid 22110, Jordan

**Keywords:** pediatric epilepsy, epilepsy treatment, perceived impact, caregiver burden, family impact, cultural factors

## Abstract

Background: Epilepsy is the most common chronic neurological condition in children, with a prevalence of 0.3% in Jordan. It significantly impacts patients’ and their families’ lives, shaped by cultural and socioeconomic factors. This study assessed the perceived impact of epilepsy on children and their families in Jordan. Method: This was a hospital-based, cross-sectional study recruiting 184 children under 18 years with epilepsy using a custom-designed questionnaire. Results: Nearly half of the patients experienced epilepsy onset before age three, and seizures were controlled in 73%. Around 60% of parents were satisfied with their understanding of the disease. Male gender and older age at diagnosis were associated with greater perceived severity, while older age at diagnosis correlated with a negative impact on caregivers’ earning potential. Patients with more anti-epileptic drugs reported more social constraints and older children expressed concerns about medication and seizure-related injuries. Cultural factors, such as family size and history, were associated with higher caregiver burden, emphasizing the importance of culturally sensitive assessment tools. Conclusions: Effective seizure control and quality of life improvements should be priorities in managing epilepsy in children. Cultural factors are strongly linked to caregiver burden, emphasizing the need for culturally sensitive assessment tools for enhancing support and care outcomes across diverse populations.

## 1. Introduction

Epilepsy, characterized by unpredictable seizures, stands as a prevalent chronic neurological disorder affecting individuals across all age spectrums, with notable peaks in both the young and elderly populations [1,2]. Its prevalence among children, ranging from 0.5% to 1%, designates it as the most common chronic neurological condition in childhood [2]. Within the Arab world, regional variances manifest with a lifetime prevalence of epilepsy reported between 2.52 and 12.67 per 1000 individuals and incidence rates spanning from 43.14 to 174.00 per 100,000 [3].

Despite the existence of global prevalence statistics, obtaining accurate figures for epilepsy within Jordan remains a challenge. Estimates from the Global Burden of Diseases 2017 suggest a prevalence of 0.3% within the country [4]. Earlier national studies highlighted deficiencies in public understanding of the condition, while a recent cross-sectional analysis revealed a moderately positive societal attitude toward epilepsy [5].

Pediatric epilepsy presents unique challenges that extend far beyond seizure control, significantly impacting multiple aspects of a child’s life and their family’s well-being. Children with epilepsy often experience comorbidities such as developmental delays, speech and language impairments, intellectual disabilities, and attention deficit hyperactivity disorder (ADHD), as well as other neurological and psychiatric conditions [6]. These comorbidities contribute to difficulties in academic performance, social interactions, and emotional well-being, further compounding the challenges faced by their families. Families often report increased stress, financial burdens, and strained interpersonal relationships as they adapt to caregiving roles [7].

Furthermore, restrictions on activities such as driving—especially during adolescence—limit the child’s independence and future opportunities, which can negatively impact their quality of life and mental health [8]. These unique challenges emphasize the importance of addressing not just the medical management of epilepsy but also its broader psychosocial effects on children and their families.

The economic burden of epilepsy is diverse, with annual total direct healthcare costs ranging from JOD 10,192 to JOD 47,862 per individual [9]. However, its impact transcends mere clinical implications, significantly influencing the perceived effect on different aspects of the life of those affected. Managing this condition notably affects social and educational achievements, as well as access to quality healthcare [10,11]. Individuals coping with epilepsy endure heightened emotional, behavioral, academic, and social challenges compared to peers with other chronic health conditions [12]. Furthermore, the stigma surrounding epilepsy inflicts self-esteem issues, depression and anxiety, and escalates the risk of suicide [13].

The perceived impact of epilepsy extends far beyond the individual diagnosed with the condition, significantly affecting their families. Studies have documented increased rates of social and marital disruptions, strained parent–child relationships, and heightened levels of stress, depression, and anxiety among families affected by epilepsy, particularly among mothers [14].

Despite its widespread global prevalence, there remains a notable lack of data detailing the psychosocial effects of epilepsy within the regional context. Hence, this study aims to explore the multifaceted impact of epilepsy on both children and their caregivers within a prominent tertiary hospital in Amman, Jordan.

In the majority of studies, patients with epilepsy have reported a lower overall quality of life [15]. However, few studies have specifically examined the impact of epilepsy on children and their caregivers’ quality of life. According to a study conducted in Saudi Arabia, the most common concerns among children and adolescents with epilepsy include initiating social relationships and concerns about how their peers at school would perceive them if they had a seizure [16].

A study in Iraq assessing stigma and psychological distress among caregivers of children with epilepsy found that the majority of participants experienced a moderate level of distress [17]. In Egypt, a negative correlation was observed between caregiver burden and quality of life, with caregivers reporting significant impairments in multiple aspects, including physical functioning, bodily pain, and emotional well-being [18]. In Jordan, a strong positive correlation was found between psychosocial burden and stigma perception among adult patients with epilepsy [19]. Additionally, a scoping review indicated that parents’ and caregivers’ fears and concerns extend beyond their child’s seizures, affecting multiple aspects of family life [20].

Assessing the perceived impact of epilepsy can provide insights into the quality of comprehensive healthcare services available to these patients and highlight additional factors that should be considered in patient care. However, research in Jordan on the psychosocial impact of epilepsy on children and their caregivers remains limited. This study seeks to address this gap and analyze its findings in an integrated manner.

## 2. Materials and Methods

This descriptive epidemiological study aimed to investigate the perceived impact of epilepsy on various aspects of life for pediatric patients and their families in Jordan. Using a cross-sectional research design, the study targeted Jordanian children under 18 years old with a confirmed epilepsy diagnosis by specialized pediatric neurologists. Participants, aged between 2 months and 18 years, were recruited from the Neurology Clinic at Prince Hamzah Hospital, a tertiary referral center in Amman. Data collection involved reviewing hospital records and conducting face-to-face interviews with children and their caregivers using a custom-designed and validated questionnaire. Epilepsy was classified based on the International League Against Epilepsy (ILAE) system into focal (partial), generalized, and combined epilepsy types. Diagnoses were confirmed by pediatric neurologists following standard clinical assessments, electroencephalography (EEG) findings, and neuroimaging results when indicated. The diagnostic criteria aligned with ICD-10 code G40, which categorizes “Epilepsy and recurrent seizures.” Although the study included children with different epilepsy subtypes, their specific impact on psychosocial outcomes was not analyzed.

Its reliability was tested with internal reliability assessments on a pilot sample of 20 questionnaires, resulting in a Cronbach’s alpha coefficient of 0.735. This questionnaire was designed to encompass a broad spectrum of inquiries, incorporating three sections. The first section has two parts, with the first part focused on sociodemographic data and family history, and the second part focused on antiseizure medication and seizure control. The first part included questions about the child’s current age, the age at diagnosis, the number of family members, the order of the epileptic child in the family, whether the child suffers from another neurological disorder or chronic disease, and the presence of a family history of epilepsy or chronic disease. The second part included questions about the number of antiseizure medication the child receives, the frequency of medication administration, and how well the seizures were controlled over the last three months.

The second section included questions regarding the perception of caregivers toward the child’s diagnosis. The level of understanding of the disease was defined as having knowledge about the etiology of epilepsy, complications of epilepsy, epilepsy medications side effects, and nonpharmacological treatments of epilepsy. Caregivers were asked how they rated the severity of the disease, frequency of seizures, duration of seizures, postictal period, or associated injuries.

Regarding the impact of epilepsy on the child, they were asked if they believed the diagnosis negatively impacted the child’s social activities or interfered with academic performance. Questions related to the impact of the child’s diagnosis on the family included its effect on recreational activities or travel plans, emotional impact such as anxiety or fear of unpredictable seizures, impact on household environment for prevention of injures, and impact on caregivers leading to conflicts regarding treatment approaches and care. Questions regarding financial strains were also included such as the caregiver’s missed work days and medical expenses. The impact of the child’s epilepsy on family social life and relationships is also examined including isolation due to unpredictability of seizures avoiding triggers and dealing with stigma and misunderstandings. Finally, caregivers were asked about difficulties they have in administering medication on time.

The last section of the questionnaire focused on the perceived impact of epilepsy and epilepsy medications from the child’s perspective including how the epilepsy affects their school performance, social relationships, and entertainment activity. It included questions regarding the child’s acceptance of and adherence to treatment schedules, anxiety about having seizure at school or in public, and fears of being injured during a seizure. Additionally, there were questions pertaining to whether the child felt stigmatized regarding the diagnoses of epilepsy, perceived as being treated differently at home or at school.

The study was approved by both the Institutional Review Board at Hashemite University (No: 1/5/2019/2020) and the Institutional Review Board at the Jordanian Ministry of Health/Prince Hamzah Hospital (No: 1/1631). Participation in the study was contingent upon the provision of voluntary written consent by all individuals involved.

Statistical analyses were executed utilizing descriptive statistics to meticulously examine the demographic, clinical, and social data collected, which were presented as frequencies (number and percentages). All variables presented in this study were categorical variables (yes/no, male/female, etc.) or Likert scale (no/yes mild/yes moderate/yes high, no/yes little/yes good/yes excellent, etc.). For the Likert scale, the answers of different degrees of “yes” were combined and used in analysis as (yes/no) crosstabulation. Furthermore, the analytical arsenal encompassed the application of Chi-square and Fisher’s exact tests to establish associations. The entire analysis process was conducted using IBM SPSS version 25.0, with the threshold for statistical significance set at ≤0.05.

## 3. Results

### 3.1. Demographics and Clinical Details of Participants’ Descriptive Statistics

A total of 184 pediatric epilepsy cases, with an average age of 6.85 ± 3.92 years, participated in the study. The cohort comprised 57.3% males and 42.7% females. About 69% of participants had a family size of 4–6 children. The epileptic child order in the family was mostly the first child (27.2%) (Table 1).

Regarding epilepsy onset and duration, nearly half (46.7%) were diagnosed between 0 and 3 years, with only 0.5% diagnosed above 12 years. Concerning epilepsy duration since diagnosis, 36.1% were diagnosed before the age of year, 23% within 1.1–2 years, 14.8% within 2.1–3 years, 7.1% within 3.1–4 years, and 19.1% for over 4 years (Table 1).

Most of the cases were well controlled on monotherapy (73.4%), while 26.6% were receiving polytherapy with 2–5 anti-epileptic drugs. Over the previous three months, half the sample was seizure free, while 34.1% reported 1–2 seizures, and 11.5% reported 3 or more seizures. Comorbidities included global developmental delay (18.0%), intellectual disability (34.2%), speech delays (25.1%), cerebral palsy (3.8%), attention deficit hyperactivity disorder (3.8%), and autism spectrum disorders (2.7%). Other neurological (6.6%) and non-neurological chronic diseases (9.8%) were also reported (Table 1).

### 3.2. Epilepsy and Its Variable Effects on Caregivers

Additionally, 82.4% of parents felt informed regarding their child’s disease, while 17.6% felt poorly informed. They rated the condition as moderate (46.4%), mild (31.17%), severe (16.4%), or not severe at all (5.5%). Furthermore, 77% believed epilepsy would adversely affect their child’s future. A total of 39.3% of them deemed the impact on their child’s future as high compared to 22.4% who deemed it moderate and 15.3% who deemed it mild. Most of the study population (79.8%) reported that the disease is not causing any familial problems compared to 20.2% who deemed the disease as impacting the familial relationship in a negative way. A total of 100 (55.5% of the total sample) reported that their child’s condition is negatively impacting the main family provider’s work while 80 (44.4%) reported no impact. Regarding leisure time and activities, almost half of the sample reported a negative impact on their child’s condition. While 74.3% think that having a patient with epilepsy in their family is not impacting their social life, 69.4% deemed this fact as a financial burden. Moreover, over half of the sample think that the commitment to timely providing medication to their children is burdensome (Table 2).

### 3.3. The Effect on the Child

Participants reported that 84.8% of children suffer from their epileptic illness. Child education was affected in 71.6% of cases. The treatment significantly affected the child’s daily activities in 75% of cases, and a similar percentage reported the child’s concern to take the prescribed medication. Caregivers reported that the children fear being hurt during seizing in 62.5% of the cases. They further reported that they sense their child’s feeling of being treated differently by their family in 55.6% of the cases. Almost two-thirds of the sample reported their child’s feeling of no preferential treatment in school. A total of 43.7% of the sample reported no impact on the child’s entertainment activities. Relationships with peers, entertainment activities, and treatment adherence were particularly affected among older children and those diagnosed later in life (Table 3).

### 3.4. Associations Between Demographics, Epilepsy, and Clinical History with Caregivers’ and Children’s Effects

Significant association was noted between the male gender and the child’s disease severity as rated by family (male 59.9% vs. female 40.1%, *p* = 0.013). A significant association was noted between increased current age and the child’s school performance (age less than 6 years 13.0% vs. age above 6 years 87.0%, *p* = 0.048), the child’s concern to take the treatment (age less than 6 years 26.0% vs. age above 6 years 74.0%, *p* = 0.005), and being fearful of being hurt during seizures (age less than 6 years 16.0% vs. age above 6 years 84.0%, *p* = 0.001). Furthermore, significant association was noted between younger age when diagnosed and family’s rating of the child’s disease severity (age less than 6 years 71.7% vs. age above 6 years 28.3%, *p* = 0.024), its impact on the family provider’s work (age less than 6 years 80.0% vs. age above 6 years 20.0%, *p* = 0.006), the child’s entertainment activities (age less than 6 years 77.6% vs. age above 6 years 22.4%, *p* = 0.047), concern about taking the treatment (age less than 6 years 56.7% vs. age above 6 years 43.3%, *p* = 0.010), and fear of injury during seizures (age less than 6 years 54.1% vs. age above 6 years 45.9%, *p* = 0.021). No statistically significant effects of epilepsy disease duration were identified on the aforementioned variables (Table 4).

A significant association was observed between large family size and the impact on the family’s social relationships (family size above three with 91.5% vs. family size less than three with 8.5%, *p* = 0.048), as well as lower birth order of the ill child and effect on the family’s daily life pattern (*p* = 0.034). Significant association was noted between higher number of seizures in the last three months and family’s leisure activities in a negative way (*p* = 0.045), the child’s entertainment activities (*p* = 0.017), the child’s concern about taking treatment (*p* = 0.006), being fearful of being hurt during seizures (*p* = 0.042), and the child feeling that they are treated differently in their school because of their disease (*p* = 0.019).

A significant association was found between a history of chronic illness in the child and the child’s relationships with other children (*p* = 0.012). Also, there was a significant association between the presence of neurological diseases other than epilepsy and perceived negative impact on the child’s future (*p* = 0.000), the family’s daily life pattern (*p* = 0.032), and increased financial burden (*p* = 0.008), the child’s suffering from their illness (*p* = 0.004), the child’s school performance (*p* = 0.000), the child’s relationship with peers (*p* = 0.000), and the child’s entertainment activities (*p* = 0.000).

There was a significant association between an increased number of anti-epileptic drugs and the family’s social relationships (*p* = 0.020), as well as with an increased financial burden (*p* = 0.035). However, the number of anti-epileptic doses per day showed no significant association with any of the variables (*p* > 0.05). Moreover, there was a significant association between positive family history of epilepsy and the family’s daily life pattern (*p* = 0.011), the child’s relationships with other children (*p* = 0.005), and the child’s entertainment activities (*p* = 0.009) (Table 4).

## 4. Discussion

This study, conducted in Jordan, utilized a custom-designed questionnaire to capture culturally embedded perceptions and behaviors in caregivers of children with epilepsy. Traditional tools often fail to consider the unique cultural contexts that caregiving experience. In Jordan, where larger family sizes and strong collectivist traditions shape caregiving, the study found a significant association between family size and the perceived impact on social relationships. This underscores the importance of understanding caregiving as a shared family experience rather than an individual one.

This study underscores the profound perceived impact of epilepsy on different aspects of life for both children and their caregivers. It illuminates various aspects of daily life affected by the condition, in agreement with prior research that emphasizes the expansive impact of epilepsy, not solely limited to affected children but extending to the entire family. It notably influences daily activities, social relationships, and financial aspects, corroborating the existing literature [21,22].

In contrast to previous studies primarily focusing on higher unemployment rates among individuals with epilepsy, this research uncovers an adverse impact on the main family provider’s income. This might stem from the well-documented burdens associated with caring for children with epilepsy, encompassing physical, economic, and social dimensions [7,23,24]. The financial strain experienced by families is a noteworthy aspect that requires attention in comprehensive epilepsy care plans.

Interestingly, older children and a later age of diagnosis were associated with a poorer perceived impact of epilepsy on life, which was also reported by Rozensztrauch et al. [21] and Nadkarni et al. [25], suggesting that children diagnosed later may perceive the disease more negatively and they find it more challenging to cope with disease complications and medication [21].

Additionally, adolescent tendencies toward independence and self-awareness may contribute to a more negative attitude toward their condition [26]. Caregivers of adult epilepsy patients experienced higher levels of burden and afflicted stigma [27], which might also be applied to older children with epilepsy. However, deeper insights into parental perspectives and the psychosocial impact on adolescents coping with epilepsy are warranted. Further exploration of the emotional and social challenges faced by older children could provide valuable insights into tailoring support mechanisms for this demographic.

In contrast to previous reports of family functioning issues, a majority of caregivers in this study reported no impact on familial relationships. This contradiction might stem from caregivers’ tendencies toward self-blame or an incomplete understanding of the situation. In addition, while the difference in lifestyle and family activity might be found according to variant cultural background, the effect of the child disease on the family can also be variable.

Furthermore, the way in which people define quality of life appears to vary significantly across cultures, as do the factors that affect the impact of different aspects of life, so paying attention to this diversity while performing cross-cultural comparable data is challenging [28].

Culture, relationships, and health are fundamental aspects of human social life. Gaining a deeper, more nuanced, and inclusive understanding of culture, which encompasses the vast diversity of human relationship experiences, can provide significant new insights into the role of relationships in health [29].

However, exploring the nuanced dynamics within families coping with epilepsy remains critical to offer comprehensive support [30,31,32].

Furthermore, the study based on qualitative findings showed that families caring for patients with epilepsy with uncontrolled seizures projected worries and uncertainty about future care provision, and seizure frequencies were found to moderate the level of caregiving burden [33].

Seizure frequency emerged as a critical factor associated with the perceived negative effect on different aspects of the patient’s life, as is consistent with previous studies. However, the impact on education, while notable (71.6%), did not reach statistical significance, potentially due to a smaller subset of school-aged children with frequent seizures in this study. The study highlights the need for further investigation into how educational institutions can better support children managing epilepsy to ensure their educational needs are met [16,34,35,36].

The study highlighted a significant impact of epilepsy on school relationships, potentially linked to stigma, misconceptions, and limited public understanding. These societal attitudes adversely affect self-esteem, leading some patients to conceal their condition. Efforts to reduce stigma and enhance public awareness are crucial to foster inclusive environments for children living with epilepsy [5,13,16,36].

The study population included children with different epilepsy subtypes, classified according to the ILAE system. While epilepsy type may influence psychosocial outcomes, the current analysis did not stratify findings by seizure classification. Future studies should explore whether specific epilepsy types have distinct psychosocial impacts on children and caregivers.

Practical implications for healthcare providers: The findings of this study highlight the need to integrate psychosocial screening into routine epilepsy care to identify caregiver stress, stigma concerns, and emotional challenges early. In addition to medical management, caregiver education programs should address misconceptions, coping strategies, and mental health support to improve family well-being. A multidisciplinary approach involving mental health professionals, social workers, and educators can provide holistic care for children with epilepsy and their families. Healthcare institutions should also promote community-based support programs and school-based epilepsy awareness initiatives to reduce stigma and improve social integration. Furthermore, healthcare providers are encouraged to adopt culturally sensitive assessment tools to better address the unique needs of families within different social and cultural contexts.

By adopting these strategies, healthcare providers can enhance the overall well-being of both patients and caregivers, ensuring that epilepsy management extends beyond medical treatment to include psychosocial and educational support.

## 5. Conclusions

This study in Jordan shows that epilepsy significantly impacts various aspects of life for both children and their families. Prioritizing seizure control, reducing frequency, and comprehensive care plans are essential to alleviate the condition’s perceived impact by enhancing independence and involving not just the child but also their family and caregivers. The study identified strong associations between cultural factors, such as family size and family history, and the psychosocial effects of epilepsy, highlighting the necessity for more culturally sensitive assessment tools.

The custom-designed questionnaire used in this study proved effective in capturing the unique experiences of caregivers in this context, making it a valuable contribution to epilepsy care research. Future studies should focus on developing and implementing culturally tailored tools to better support caregivers and improve care outcomes across diverse populations.

## 6. Strengths and Limitations

Strengths of the study include the in-depth analysis of family perceptions of the effect on different aspects of the life of children with epilepsy, though there might be parental concerns affecting reported impacts. Limitations include a lack of consideration for socioeconomic status, potential sampling bias due to convenience sampling, and the absence of a standardized tool for comparisons across countries. Participants were recruited exclusively from the Neurology Clinic at Prince Hamza Hospital, a tertiary referral center in Amman that serves patients from various geographic areas in Jordan. While this enhances diversity, single-site recruitment may still introduce selection bias and limit the generalizability of findings to patients outside this setting.

The questionnaire used is self-designed and not previously used, and the available questionnaires are culturally biased and were not translated according to cultural appropriate modification. Maintaining sensitivity to cross-cultural diversity while comparing data is challenging.

Understanding the diverse cultural foundations of human relationships is crucial for gaining insights into their role in health. This requires acknowledging cultural influences and systematically including diverse perspectives to integrate cultural studies into the examination of relationships and health.

## Figures and Tables

**Table 1:** Demographic and clinical data of study participants (*n* = 184).

Category	Variable		Number (Percentage %)
Demographics	Age (years)	0–3	42 (23)
		3.1–6	43 (23.5)
		6.1–9	45 (24.6)
		9.1–12	36 (19.7)
		Above 12	17 (9.3)
	Gender	Male	102 (57.3)
		Female	76 (42.7)
	Family size	0–3	14 (7.6)
		4–6	126 (68.9)
		7 or more	43 (23.5)
	Ill child’s order in the family	First	50 (27.2)
		Second	46 (25.0)
		Third	36 (19.6)
		Fourth	30 (16.3)
		Fifth or more	22 (11.9)
Epilepsy onset and duration	Age when diagnosed (years)	0–3	86 (46.7)
		3.1–6	45 (24.5)
		6.1–9	38 (20.7)
		9.1–12	14 (7.6)
		Above 12	1 (0.5)
	Duration	0–1 years	66 (36.1)
		1.1–2 years	42 (23.0)
		2.1–3 years	27 (14.8)
		3.1–4 years	13 (7.1)
		More than 4 years	35 (19.1)
Symptoms	Seizure frequency in the last 3 months		
	<once monthly		136 (73.9)
	1–10/month		32 (17.4)
	10+/month		14 (7.6)
Neurological diseases (total 98)	Developmental delay		33 (18.0)
	Learning difficulties		63 (34.2)
	Delayed speech		46 (25.1)
	Cerebral palsy		7 (3.8)
	ADHD		7 (3.8)
	Autism		5 (2.7)
	Others		12 (6.6)
Chronic diseases (total 31)	Diabetes		3 (1.6)
	Asthma		7 (3.8)
	Phenylketonuria		1 (0.5)
	Hypothyroidism		2 (1.1)
	Familial Mediterranean Fever		1 (0.5)
	Thalassemia		1 (0.5)
	Others		18 (9.8)
Family history	Epilepsy		79 (43.2)
	First degree		19 (10.3)
	Second degree		36 (19.6)
	Others		24 (13.1)
	Hypertension		28 (15.3)
	Diabetes		20 (10.9)
	Hypothyroidism		6 (3.3)
	Asthma		6 (3.3)
Anti-epileptic	Number of drugs		
	One		135 (73.4)
	Two		40 (21.7)
	Three or more		9 (4.9)
	Number of doses per day		
	One		10 (5.5)
	Two		167 (92.3)
	Three or more		4 (2.2)

**Table 2:** Effect of child epilepsy on caregivers.

Category	Variable	Number (Percentage %)
Do you think you have sufficient understanding of your child disease?	yes, excellent understanding	108 (59.3)
	yes, good understanding	42 (23.1)
	yes, little understanding	8 (4.4)
	no, not at all	24 (13.2)
How would you rate your child’s disease severity?	very severe	30 (16.4)
	moderate severity	85 (46.4)
	mild severity	58 (31.7)
	not severe	10 (5.5)
Do you think that your child’s disease will have negative impact on his future?	yes, high negative impact	72 (39.3)
	yes, moderate negative impact	41 (22.4)
	yes, mild negative impact	28 (15.3)
	no impact at all	42 (23.0)
Does your child’s disease affect the family’s daily life pattern?	yes, high impact	75 (41.2)
	yes, moderate impact	35 (19.2)
	yes, mild impact	15 (8.2)
	no impact at all	57 (31.3)
Does your child’s disease causes any problems within the family?	yes, highly	18 (9.8)
	yes, moderately	7 (3.8)
	yes, mildly	12 (6.6)
	no	146 (79.8)
Does your child’s disease affect the family provider work? (in a negative way)	yes, highly	52 (28.9)
	yes, moderately	13 (7.2)
	yes, mildly	35 (19.4)
	no	80 (44.4)
Does your child’s disease affect the family’s leisure activities (in a negative way)?	yes, highly	43 (23.5)
	yes, moderately	9 (4.9)
	yes, mildly	38 (20.8)
	no	92 (50.3)
Does your child’s disease affect the family’s social relationships?	yes, highly	17 (9.3)
	yes, moderately	12 (6.6)
	yes, mildly	18 (9.8)
	no	17 (74.3)
Does your child’s disease causes more financial burden?	yes, highly	63 (34.4)
	yes, moderately	18 (9.8)
	yes, mildly	46 (25.1)
	no	56 (30.6)
Do you have problems in commitment to give your child his drug at specific times?	yes, highly	31 (16.9)
	yes, moderately	27 (14.8)
	yes, mildly	38 (20.8)
	no	87 (47.5)

**Table 3:** The effect of child epilepsy on the child themself.

Category	Variable	Number (Percentage %)
How much the child suffer from his own disease?	very	57 (36.1)
	mild	49 (31.0)
	little	28 (17.7)
	not at all	24 (15.2)
How much the disease affects the child’s school performance?	very	53 (48.6)
	mild	14 (12.8)
	little	11 (10.1)
	not at all	31 (28.4)
How much the disease affects the child’s relationships with other children?	very	21 (13.1)
	mild	28 (17.5)
	little	32 (20.0)
	not at all	79 (49.4)
How much the disease affects the child’s entertainment activities	very	22 (12.6)
	mild	33 (19.0)
	little	43 (24.7)
	not at all	76 (43.7)
How much the treatment affect the daily activities of the child?	very	36 (19.8)
	mild	43 (23.6)
	little	55 (30.2)
	not at all	48 (26.4)
How concern is your child to take the treatment?	very	64 (44.8)
	mild	21(14.7)
	little	19 (13.3)
	not at all	39 (27.3)
How fearful is your child of getting hurt during seizures?	very	38 (27.9)
	mild	13 (9.6)
	little	34 (25.0)
	not at all	51 (37.5)
Does the child feel that he is treated differently by his family members, because of his disease?	yes, highly	55 (38.2)
	yes, moderately	5 (3.5)
	yes, mildly	20 (13.9)
	no	64 (44.4)
Does the child feel that he is treated differently in his school, because of his disease?	yes, highly	18 (17.3)
	yes, moderately	8 (7.7)
	yes, mildly	11 (10.6)
	no	67 (64.4)

**Table 4:** Associations between age, gender, disease duration, family size, ill child’s order, number of seizures, chronic diseases, anti-epileptic treatment, and family history of epilepsy with effects on caregivers and children.

Family History Epilepsy	Number of Doses	Number of Anti-Epileptic	Neurological Diseases	Chronic Diseases	Number of Seizures	Child Order	Family Size	Duration	Age at Diagnosis	Age Now	Gender	
0.552	0.437	0.374	0.685	0.979	0.855	0.505	0.513	0.987	0.764	0.449	0.717	Do you think you have sufficient understanding of your child disease?
0.823	0.223	0.675	0.125	0.574	0.128	0.593	0.309	0.843	0.024	0.243	0.013	How would you rate your child’s disease severity?
0.785	0.626	0.113	0.000	0.674	0.069	0.456	0.715	0.295	0.322	0.790	0.667	Do you think that your child’s disease will have negative impact on his future?
0.011	0.125	0.606	0.032	0.636	0.265	0.034	0.850	0.117	0.415	0.341	0.257	Does your child’s disease affect the family’s daily life pattern?
0.814	0.653	0.739	0.420	0.336	0.164	0.609	0.288	0.448	0.089	0.311	0.751	Does your child’s disease causes any problems within the family?
0.639	0.713	0.736	0.205	0.650	0.230	0.454	0.623	0.559	0.006	0.134	0.848	Does your child’s disease affect the family provider work? (in a negative way)
0.780	0.060	0.225	0.259	0.090	0.045	0.889	0.071	0.084	0.311	0.323	0.295	Does your child’s disease affect the family’s leisure activities (in a negative way)?
0.838	0.685	0.020	0.101	0.294	0.085	0.079	0.048	0.500	0.222	0.415	0.605	Does your child’s disease affect the family’s social relationships?
0.455	0.742	0.035	0.008	0.826	0.238	0.522	0.612	0.426	0.897	0.910	0.523	Does your child’s disease causes more financial burden?
0.921	0.601	0.355	0.287	0.918	0.898	0.751	0.373	0.541	0.623	0.568	0.268	Do you have problems in commitment to give your child his drug at specific times?
0.727	0.871	0.720	0.004	0.901	0.453	0.818	0.830	0.070	0.572	0.475	0.850	How much the child suffer from his own disease?
0.690	0.670	0.403	0.000	0.283	0.736	0.444	0.260	0.368	0.783	0.048	0.108	How much the disease affects the child’s school performance?
0.005	0.548	0.101	0.000	0.012	0.055	0.239	0.701	0.440	0.850	0.306	0.929	How much the disease affects the child’s relationships with other children?
0.009	0.627	0.285	0.000	0.097	0.017	0.255	0.440	0.912	0.047	0.596	0.991	How much the disease affects the child’s entertainment activities
0.086	0.721	0.248	0.060	0.155	0.304	0.498	0.064	0.533	0.887	0.773	0.992	How much the treatment affect the daily activities of the child?
0.066	0.536	0.336	0.060	0.377	0.006	0.442	0.307	0.692	0.010	0.005	0.200	How concern is your child to take the treatment?
0.199	0.291	0.867	0.120	0.668	0.042	0.098	0.527	0.438	0.021	0.001	0.818	How fearful is your child of getting hurt during seizures?
0.255	0.539	0.523	0.575	0.549	0.078	0.457	0.192	0.912	0.097	0.216	0.807	Does the child feel that he is treated differently by his family members, because of his disease?
0.165	0.474	0.948	0.198	0.598	0.019	0.200	0.536	0.967	0.516	0.357	0.511	Does the child feel that he is treated differently in his school, because of his disease?

## Data Availability

The original contributions presented in the study are included in the article.
